# 한국 성인 음주 남성의 고위험 음주 관련 요인: 2차 분석 연구

**DOI:** 10.7586/jkbns.23.026

**Published:** 2024-02-21

**Authors:** Hyun Ju Chae

**Affiliations:** Department of Nursing, Sangmyung University, Cheonan, Korea; 상명대학교 간호학과

**Keywords:** Adult, Men, Alcohol drinking, Health risk behaviors, 성인, 남성, 음주, 건강위험행동

## Abstract

**Purpose:**

This study was conducted to identify factors associated with high-risk drinking in Korean men.

**Methods:**

This study was a secondary analysis using data from the Korea National Health and Nutrition Examination Survey (KNHANES VIII-2), 2020. Data were downloaded from the KNHANES website. The subjects of this study were 1,653 alcohol-drinking men between 19 and 64 years of age. Data were analyzed using the Rao-Scott chi-square test and complex sample logistic regression statistics.

**Results:**

The high-risk drinking rate among alcohol-drinking men was 27.1%. High-risk drinking was more common in men who smoked (odds ratio [OR] = 2.11, *p* < .001), men with a middle school education or lower (OR = 1.91, *p* = .016), men who lived with a spouse (OR = 1.61, *p* = .025), men who slept less than 6 hours on weekends (OR = 1.51, *p* = .016), and men who perceived stress (OR = 1.30, *p* = .044), while it was lower in men who were underweight (OR = 0.19, *p* = .006).

**Conclusion:**

To reduce the rate of high-risk drinking, it is necessary to provide an intervention that considers factors associated with high-risk drinking among men. In particular, smoking was the most important factor associated with high-risk drinking, implying that the integrated management of drinking and smoking is necessary to reduce high-risk drinking.

## 서론

### 1. 연구의 필요성

음주는 문화, 종교, 및 사회적 관행의 일부로 사회에 깊숙이 자리잡고 있으며, 전 세계적으로 약 23억명의 사람들이 음주를 하고 있다[1,2]. 적당한 양의 음주는 기분을 좋게 하고 긴장을 완화시켜 대인관계에 있어 윤활제 역할을 하기도 한다[2]. 그러나 일정 수준 이상의 과도한 음주는 다양한 문제를 초래한다. 음주는 알코올의존, 간경화증, 당뇨병, 심혈관질환, 암 등과 같은 만성질환의 주요 위험요인이며[3], 에이즈, 결핵, 바이러스간염 및 성매개감염과 같은 전염병의 질병 부담에 기여한다는 증거도 점점 늘어나고 있다[2]. 음주로 인한 사망률은 결핵, 당뇨병, 에이즈와 같은 질병으로 인한 사망률보다 높은데 2016년 전 세계적으로 약 300만명(전체 사망의 5.3%)이 음주로 인해 사망한 것으로 보고되었으며, 음주로 인한 사망 중 28.7%는 부상, 21.3%는 소화기질환, 19%는 심혈관질환, 12.9%는 전염병, 12.6%는 암으로 인한 것으로 나타났다[2]. 또한 음주로 인한 부상 및 질병은 기대수명을 단축시키는 것으로 보고되고 있다[3]. 음주는 개인의 건강뿐만 아니라 간접적이고 이차적인 영향을 통해 사회전반에 위험을 증가시키는 중요한 보건학적 문제이다[4]. 음주는 가족, 친구, 동료, 낯선 사람 등 다른 사람에게도 해를 끼칠 수 있는데, 음주운전으로 인한 교통사고나 주취 폭력 등으로 인해 타인에게 부상이나 손상을 초래하고 괴롭힘, 모욕, 위협과 같은 문제도 초래할 수 있다[1,2,4]. 또한 의료비 상승, 생산력 손실, 실업 비용 증가, 사고나 폭력으로 인한 사법비용 증가 등과 같은 사회경제적 손실과 부담을 증가시킨다[1,5].

고위험 음주는 건강위험행동으로 다양한 수준의 신체적, 심리적, 행동적 문제를 경험하게 만드는 수준의 음주를 의미한다[4]. 고위험 음주에 대한 정의와 기준은 다양한데[5], 세계보건기구(World Health Organization, WHO)는 지난 30일 동안 60g 이상의 순수 알코올을 1회 이상 소비한 경우로 정의하고 있으며[1], 우리나라는 국민건강영양조사에서 1회 평균 음주량이 남성은 7잔 이상, 여성은 5잔 이상이면서 일주일에 2회 이상 음주하는 경우를 고위험 음주로 정의하고 있다[6]. 우리나라의 경우 음주에 대해 관대한 문화적 특성이 있어 다른 나라들보다 알코올 섭취량과 고위험 음주 비율이 높게 나타나고 있다[4,7]. 2018년 WHO의 알코올과 건강에 대한 보고서에서 우리나라 15세 이상 남녀 1인당 알코올 섭취량은 10.2L로 WHO 평균 6.4L에 비해 높고, 15세 이상 남녀의 고위험 음주 비율은 30.5%로 WHO 평균 18.2%에 비해 높은 것으로 나타났다[2]. 성별에 따라 파악해 보면, 15세 이상 1인당 알코올 섭취량은 여성 3.9L, 남성 16.7L로 여성은 WHO 평균보다 낮은 반면 남성은 WHO 평균보다 2.6배 높고, 15세 이상 고위험 음주 비율도 여성 13.6%, 남성 47.8%로 여성은 WHO 평균보다 낮은 반면 남성은 WHO 평균보다 2.6배 높게 나타났다[2]. 이는 우리나라의 알코올 섭취량과 고위험 음주율이 높게 나타난 것은 남성의 알코올 섭취량과 고위험 음주율이 높기 때문임을 보여주는 것으로 남성의 고위험 음주를 낮추기 위한 대책 마련의 필요성을 보여주는 것이라고 할 것이다. 또한 코로나바이러스병-19(코로나19) 팬데믹 기간 동안 전 세계적으로 음주량이 증가한 것으로 나타났고[8-10], 우리나라의 경우도 코로나19 팬데믹 이후 성인 남성의 고위험 음주율이 큰 폭으로 증가한 것으로 나타나[11], 남성의 고위험 음주를 낮추기 위한 대책 마련은 시급한 과제라고 할 수 있다.

음주로 인한 건강 및 사회적 부담은 대부분 예방할 수 있기 때문에 WHO에서는 음주로 인한 폐해를 줄이기 위한 정책 지침을 제공하고 있다[1]. 우리나라도 제4차 국민건강증진종합계획의 절주 분야를 통해 음주 관련 대책을 수립하고 추진하였으나 실행은 미흡한 상황으로[12]. 우리나라의 음주 관련 정책 수준은 OECD 국가 중 최하위 수준이다[4]. 음주 관련 성과를 도출하기 위해서는 실질적인 행동계획을 수립하고 추진하는 것이 필요하며[12], 이러한 행동계획 수립을 위한 근거를 제공하기 위해 지속적인 연구가 수행될 필요가 있다. 고위험 음주와 관련된 요인에 대한 선행연구들에서 성별, 교육수준, 직업과 같은 인구사회학적 요인들과 흡연, 스트레스, 우울, 비만, 주관적 건강상태와 같은 건강요인들이 고위험 음주와 관련 있음을 보고하고 있다[5]. 그러나 남성의 고위험 음주가 시급한 문제이므로 이에 대한 연구가 필요하나 남성만을 대상으로 고위험 음주와 관련된 요인을 파악하는 연구는 부족하며[13], 코로나19 팬데믹 이후 남성의 고위험 음주와 관련된 요인에 대한 연구는 없는 실정이다. 이에 본 연구에서는 코로나19 팬데믹 이후 성인 음주 남성의 고위험 음주 관련 요인을 파악하여 남성의 고위험 음주를 낮추기 위한 정책 수립에 대한 기초자료를 제공하고자 한다.

### 2. 연구 목적

본 연구는 성인 음주 남성의 일반적 특성과 건강 관련 특성 및 건강행태에 따른 고위험 음주율의 차이를 분석하고, 성인 음주 남성의 고위험 음주 관련 요인을 파악하기 위해 수행되었다.

## 연구 방법

### 1. 연구 설계

본 연구는 코로나19 팬데믹 후 우리나라 성인 음주 남성의 고위험 음주 관련 요인을 파악하기 위해 코로나19 팬데믹 이후인 2020년에 실시한 국민건강영양조사 제 8기 2차년도 자료를 이용한 이차 분석 연구이다.

### 2. 연구 대상

**Figure 1.**
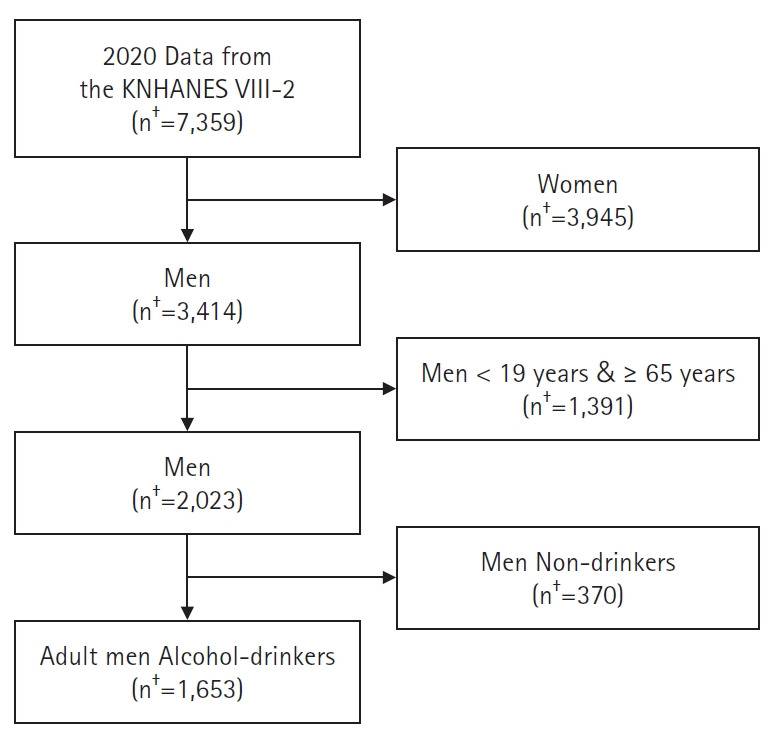
Flowchart of the selection of study subjects. KNHANES = Korea National Health and Nutritional Examination Survey. ^†^Unweighted frequency.

본 연구의 연구대상은 2020년 1월에서 12월까지 실시한 국민건강영양조사에 참여한 19~64세 성인 남성 중 음주 경험이 있는 남성을 대상으로 하였다. 음주 경험이 있는 남성은 최근 1년 동안의 음주에 대한 질문인 “술을 얼마나 자주 마십니까?”의 음주 빈도에 응답한 남성을 의미하며, 전혀 마시지 않았다고 응답한 남성은 제외하였다. 또한 고위험 음주는 한 번에 마시는 음주량과 음주 빈도에 대한 응답을 이용하여 고위험 음주 기준[6]에 따라 한 번에 마시는 음주량이 7잔 이상이고 음주 빈도가 주 2회 이상이라고 응답한 남성을 의미한다. 2020년에 실시한 국민건강영양조사 참여자는 총 7,359명으로, 남성은 3,414명이었고, 19-64세 성인 남성은 2,023명이었으며, 19-64세 성인 남성 중 음주 경험이 있는 남성은 1,653명이었다(Figure 1).

### 3. 연구 도구

본 연구에서 일반적 특성과 건강 관련 특성 및 건강행태는 국민건강영양조사의 건강설문조사와 검진 자료를 사용하였으며, 국민건강영양조사 자료를 그대로 사용하거나 본 연구 목적에 맞게 재분류하여 사용하였다.

#### 1) 일반적 특성

일반적 특성은 건강설문조사 중 만 나이, 가구 총소득, 교육 수준 재분류, 결혼 여부 및 결혼상태, 직업 재분류, 가구원 수 및 가구주와의 관계에 대한 자료를 사용하였다. 나이는 만 나이를 기준으로 19-29세, 30-39세, 40-49세, 50-59세, 60-64세로 분류하였다. 소득은 월평균 가구균등화소득에 따라 가구 총소득을 하, 중하, 중상, 상으로 4등분한 가구 총소득 자료를 그대로 사용하였고, 교육 수준은 교육 수준 재분류 자료를 이용하여 중졸 이하(초졸 이하, 중졸), 고졸, 대졸 이상으로 분류하였다. 결혼상태는 결혼 여부 및 결혼상태 자료를 이용하여 미혼, 배우자와 동거, 별거/사별/이혼으로 분류하였다. 직업은 직업 재분류 자료를 이용하여 직업 없음과 직업 있음으로 분류하였다. 세대 유형은 가구원 수 자료를 이용하여 가구원 수 1명은 일인 가구, 나머지는 다인 가구로 분류하였으며, 가구주 여부는 가구주와의 관계 자료를 이용하여 가구주가 본인인 경우는 예, 나머지는 아니오로 분류하였다.

#### 2) 건강 관련 특성

건강 관련 특성은 검진조사 및 건강설문조사 자료를 사용하였다. 검진조사 자료에서는 키와 몸무게, 허리둘레 자료를 사용하였다. 건강설문조사 자료에서는 주관적 건강인지, 평소 스트레스 인지 정도, 건강검진 및 암검진 수진, 인플루엔자 예방접종, 필요 의료서비스 미충족 자료를 사용하였다. 체질량지수는 검진조사 자료의 키와 몸무게를 이용하여 산출(kg/m^2^)하였으며, 18.5 kg/m^2^ 미만이면 저체중, 18.5 kg/m^2^ 이상~23 kg/m^2^ 미만이면 정상, 23 kg/m^2^ 이상~25 kg/m^2^ 미만이면 과체중, 25 kg/m^2^ 이상이면 비만으로 분류하였다[14]. 복부비만은 검진조사 자료의 허리둘레를 이용하여 90 cm 이상은 복부비만, 90 cm 미만은 복부비만 아님으로 분류하였다[14]. 주관적 건강 상태는 주관적 건강인지 자료를 선행연구[15]를 참고하여 좋음(매우 좋음, 좋음), 보통(보통), 나쁨(나쁨, 매우 나쁨)으로 분류하였다. 스트레스 인지는 평소 스트레스 인지 정도 자료를 선행연구[15]를 참고하여 스트레스 인지(대단히 많이 느낀다, 많이 느끼는 편이다)와 스트레스 비인지(조금 느끼는 편이다, 거의 느끼지 않는다)로 분류하였다. 건강검진·암검진·인플루엔자접종은 건강검진·암검진·인플루엔자접종 여부를 예와 아니오로 분류하였다. 미충족 의료는 최근 1년 동안 병의원 진료(검사 또는 치료)가 필요하였으나 받지 못한 경험이 있으면 미충족 의료 있음, 없으면 미충족 의료 없음으로 분류하였다.

#### 3) 건강행태

건강행태는 건강설문조사 자료 중 현재 흡연, 유산소 신체활동 실천율, 1주일 동안 걷기 일수, 1주일 동안 근력운동 일수, 평소 하루 앉아서 보내는 시간, 주중·주말 하루 평균 수면시간 자료를 사용하였다.

흡연은 현재 흡연 자료를 이용하여 흡연(매일 피움, 가끔 피움)과 비흡연(피우지 않음, 과거엔 피웠으나 현재 피우지 않음)으로 분류하였다. 신체활동은 유산소 신체활동 실천율 자료를 이용하여 일주일 동안 중강도의 신체활동을 2시간 30분 이상 실천했거나, 고강도의 신체활동을 1시간 15분 이상 실천했거나, 중강도와 고강도의 신체활동을 섞어서 각각의 활동에 상당하는 시간을 실천한 경우는 예, 나머지는 아니오로 분류하였다. 걷기와 근력운동은 1주일간 걷기 일수와 근력운동 일수 자료를 이용하였다. WHO는 성인은 주 5회 이상의 유산소운동과 주 2회 이상의 근력운동을 권고하고 있으므로[16], 1주일간 걷기 일수가 5일 미만이면 걷기 비실천, 5일 이상이면 걷기 실천으로 분류하였고, 1주일간 근력운동 일수가 2일 미만이면 근력운동 비실천, 2일 이상이면 근력운동 실천으로 분류하였다. 앉아서 보내는 시간은 평소 하루 앉아서 보내는 시간 자료를 선행연구[17]를 참고하여 10시간 미만과 10시간 이상으로 분류하였다. 주중·주말 수면시간은 주중(또는 일하는 날)과 주말(또는 일하지 않는 날, 일하지 않는 전날) 수면시간 자료를 선행연구[18]를 참고하여, 6시간 이하, 7~8시간, 9시간 이상으로 분류하였다.

### 4. 자료 수집

본 연구의 자료는 국민건강영양조사 홈페이지의 원시자료 data base (DB)에서 2020년 기본 DB 자료를 다운받은 후 사용하였다. 원시자료의 기본 DB는 검진조사 건강설문조사, 영양조사로 구성되어 있으며, 본 연구에서는 원시자료의 기본 DB 중 검진조사와 건강설문조사 자료를 사용하였다. 국민건강영양조사에서 검진조사와 건강설문조사는 이동검진 차량에서 실시하였다. 검진조사는 직접 계측방법으로 수행하였으며, 키는 0.1 cm 단위로 측정 가능한 키 측정기를 이용하여 측정하였고, 몸무게는 0.1 kg 단위로 측정가능한 이동식 디지털 몸무게 측정기를 이용하여 측정하였다. 허리둘레는 0.1 cm 간격으로 측정되는 줄자를 이용하여 측정하였다. 신체 계측 시 옷을 탈의하고 일회용 검진 가운을 착용하게 하였으며, 머리핀과 머리끈 등의 장식을 제거하고 신발과 양말 등을 벗은 상태에서 키와 몸무게를 측정하였고, 허리둘레는 좌우 측면의 장골 상단과 늑골 하단의 중간 지점을 표시하고 수평으로 연결하여 측정하였다. 건강설문조사는 면접방법으로 조사하였으며 건강설문조사 항목 중 음주, 흡연 등 건강행태와 관련된 영역은 자기기입식으로 조사하였다.

### 5. 자료 분석

본 연구에서 자료분석은 SPSS 21.0 프로그램(IBM Corp., Armonk, NY, USA)을 이용하여 분석하였다. 국민건강영양조사는 표본 추출 시 2단계의 층화집락 표본설계를 이용하였으므로, 자료분석은 층, 집락, 가중치 등의 복합표본요소를 고려한 복합표본분석법을 사용하였다. 구체적인 자료분석 방법은 다음과 같다.

1) 성인 음주 남성의 고위험 음주율은 복합표본 빈도분석을 실시하였다.

2) 성인 음주 남성의 일반적 특성과 건강 관련 특성 및 건강행태에 따른 고위험 음주율의 차이 분석은 Rao-Scott chi-square test를 실시하였다.

3) 성인 음주 남성의 고위험 음주 관련 요인은 복합표본 로지스틱 회귀분석을 실시하였다.

4) 유의수준은 *p* < .05를 기준으로 판단하였다.

### 6. 윤리적 고려

국민건강영양조사는 원시자료 공개 등을 통해 조사 결과를 공표하고 있으며, 자료 공개 시 개인을 추정할 수 없도록 개인정보보호법과 통계법에 따라 비식별화 조치된 자료만을 제공하고 있다. 본 연구를 위한 자료는 국민건강영양조사 홈페이지에 제시된 자료 이용 절차를 준수하여 통계자료 이용자 준수사항 이행 서약서를 작성하고 사용자 정보등록을 완료한 후 파일로 다운받아 사용하였다. 또한 본 연구는 중부대학교 생명윤리심의위원회에서 심의를 받아 승인 후 시행하였다(IRB No. JIRB-2023012501-01).

## 연구 결과

### 1. 성인 음주 남성의 고위험 음주율 및 일반적 특성에 따른 고위험 음주율의 차이

성인 음주 남성의 고위험 음주율은 27.1%이었으며, 연령, 교육 수준, 결혼 상태, 직업 유무, 및 가구주 여부에 따라 차이가 있었다. 연령에 따른 차이에서 19-29세 남성에서 고위험 음주율이 가장 낮게 나타났으며, 40-49세 남성에서 고위험 음주율이 가장 높게 나타났다(χ^2^ = 38.45, *p* < .001). 교육 수준이 대졸 이상인 남성에서 고위험 음주율이 가장 낮게 나타났으며, 중졸 이하인 남성에서 고위험 음주율이 가장 높게 나타났다(χ^2^ = 13.99, *p* = .006). 결혼 상태에 따른 차이는 미혼인 남성에서 고위험 음주율이 가장 낮게 나타났고, 이혼, 별거 또는 사별인 남성에서 고위험 음주율이 가장 높게 나타났다(χ^2^ = 35.15, *p* < .001). 직업이 없는 남성에 비해 직업이 있는 남성에서 고위험 음주율이 높게 나타났으며(χ^2^ = 17.74, *p* < .001), 가구주가 아닌 남성에 비해 가구주인 남성에서 고위험 음주율이 높게 나타났다(χ^2^ = 13.46, *p* = .001)(Table 1).

### 2. 성인 음주 남성의 건강 관련 특성 및 건강행태에 따른 고위험 음주율의 차이

성인 음주 남성의 고위험 음주율은 체질량지수, 복부비만 여부, 주관적 건강 상태, 및 스트레스 인지, 흡연 여부, 걷기와 근력운동 실천 여부, 좌식시간 및 주말 수면시간에 따라 차이가 있었다. 체질량지수가 저체중인 남성에서 고위험 음주율이 가장 낮고 체질량지수가 비만인 남성에서 고위험 음주율이 가장 높게 나타났으며(χ^2^ = 8.73, *p* = .036), 복부비만이 아닌 남성에 비해 복부비만인 남성에서 고위험 음주율이 높게 나타났다(χ^2^ = 8.30, *p* = .021). 주관적 건강상태를 좋음으로 인지한 남성에서 고위험 음주율이 가장 낮게 나타났고(χ^2^ = 14.65, *p* = .001), 스트레스 비인지 남성에 비해 스트레스를 인지하는 남성에서 고위험 음주율이 높게 나타났다(χ^2^ = 7.12, *p* = .005). 흡연 여부에서는 비흡연 남성에 비해 흡연 남성에서 고위험 음주율이 높게 나타났다(χ^2^ = 44.16, *p* < .001). 걷기 실천 남성에 비해 걷기 비실천 남성에서 고위험 음주율이 높게 나타났고(χ^2^ = 8.09, *p* = .016), 근력운동 실천 남성에 비해 근력운동 비실천 남성에서 고위험 음주율이 높게 나타났다(χ^2^ = 7.76, *p* = .010). 하루 평균 앉아서 보내는 시간이 10시간 이상인 남성에 비해 10시간 미만인 남성에서 고위험 음주율이 높게 나타났고(χ^2^ = 5.47, *p* = .041), 주말 평균 수면시간이 6시간 이하인 남성에서 고위험 음주율이 가장 높게 나타났다(χ^2^ = 13.67, *p* = .013) (Table 2).

### 3. 성인 음주 남성의 고위험 음주 관련 요인

성인 음주 남성의 고위험 음주는 흡연 여부, 교육 수준, 결혼 상태, 주말 수면시간, 스트레스 인지 및 체질량지수와 관련이 있었다. 비흡연 남성에 비해 흡연 남성에서 고위험 음주가 2.11배 많았고(*p* < .001), 대졸 이상인 남성에 비해 중졸 이하 남성에서 고위험 음주가 1.91배 많았다(*p* = .016), 미혼 남성에 비해 배우자와 동거하는 기혼 남성에서 고위험 음주가 1.61배 많았고(*p* = .025), 주말 수면시간이 7-8시간인 남성에 비해 6시간 이하인 남성에서 고위험 음주가 1.51배 많았으며(*p* = .016), 스트레스 비인지 남성에 비해 스트레스 인지 남성에서 고위험 음주가 1.3배 많았다(*p* = .044). 반면, 체질량지수가 저체중인 남성은 체질량지수가 정상인 남성에 비해 고위험 음주가 0.19배 적었다(*p* = .006) (Table 3).

## 논의

본 연구에서 성인 음주 남성의 고위험 음주는 비흡연 남성에 비해 흡연 남성에서 2.11배 많은 것으로 나타났다. 이는 비흡연자에 비해 흡연자에서 고위험 음주 위험이 높게 나타남을 보고한 선행연구[5,13,19]와 일치하는 결과이다. 흡연자에서 고위험 음주가 높게 나타나는 것은 국내뿐만 아니라 국외 연구에서도 일치하는 결과를 보고하고 있는데[20,21], 이러한 선행연구 결과들 및 본 연구결과를 종합하면 흡연은 고위험 음주의 중요한 예측요인이라고 할 수 있다. 따라서 고위험 음주를 감소시키기 위해서는 흡연과 음주를 동시에 관리하는 것이 필요하며[5,13,19], 코로나19 팬데믹 기간 동안 스트레스 대응 전략으로 가장 많이 사용한 것이 음주이며 다음으로 많이 사용한 것이 흡연임을 고려하면[8], 흡연과 음주를 동시에 관리하는 것은 코로나19 이후 고위험 음주 감소를 위한 필수 사항이라고 할 수 있다. 흡연과 음주를 동시에 관리하기 위해서는 현재 이원화되어 알코올 상담 센터와 보건소의 금연 사업을 일원화하는 것이 필요하며[22], 국가적 차원에서 음주와 흡연을 통합하여 관리하는 정책적 기반을 수립하고 이를 토대로 하여 실질적이고 효과적인 중재 전략을 제공하는 것이 필요하다. 또한 고위험 음주자의 흡연 상태와 흡연 정도를 파악하여[13,19], 고위험 음주자이면서 흡연을 하는 사람과 고위험 음주자이면서 흡연을 하지 않는 사람의 차이를 비교분석하고[23], 이에 따른 중재를 제공하는 것도 필요하다고 할 것이다. 또한 고위험 음주는 흡연뿐 아니라 다른 물질 사용과도 관련 있는 것으로 보고되고 있으므로[24], 고위험 음주자를 대상으로 물질 남용 예방 교육을 실시하고, 물질 남용 실태를 파악하여 이에 따른 중재를 제공하는 것도 필요하다고 할 것이다.

본 연구에서 성인 음주 남성의 고위험 음주는 대졸 이상인 남성에 비해 중졸 이하 남성에서 1.91배 많은 것으로 나타났다. 이는 교육 수준이 낮을수록 고위험 음주가 높게 나타남을 보고한 선행연구[22,23,25]와 일치하는 결과이다. 그러나 교육 수준과 고위험 음주는 관계가 없음을 보고한 선행연구[13,26,27] 및 교육 수준과 고위험 음주의 관계는 연령대로 다르게 나타남을 보고한 선행연구[5]와는 차이가 있다. 교육 수준이 낮을수록 고위험 음주가 높은 것은 교육 수준이 낮을수록 음주의 위험성에 대한 인식이 낮아 고위험 음주를 더 많이 하기 때문이라고 하였다[7]. 그러나 교육 수준은 직업과 관련이 있고[28], 단순노무직이나 판매 서비스직 종사자는 전문직 종사자에 비해 고위험 음주가 많음을 보고한 선행연구[29]를 고려하면, 교육 수준이 낮을수록 고위험 음주가 높게 나타난 것은 교육 수준에 따른 직업적 특성에 의한 결과일 수 있다. 따라서 고위험 음주를 낮추기 위해서는 교육 수준에 따른 고위험 음주 위험성에 대한 인식 정도를 파악하여 이에 따른 교육적 중재를 기본적으로 제공하고, 교육 수준에 의해 영향을 받을 수 있는 직업 특성 등의 사회경제적 요인을 함께 고려한 중재를 추가하는 것이 필요하다고 할 것이다.

본 연구에서 성인 음주 남성의 고위험 음주는 미혼 남성에 비해 배우자와 동거하는 기혼 남성에서 1.61배 많은 것으로 나타났다. 이는 미혼 남성에 비해 사별, 별거, 이혼, 및 기혼 남성에서 고위험 음주가 높게 나타남을 보고한 선행연구[23]와 유사한 결과이다. 그러나 결혼 상태와 고위험 음주는 관계가 없음을 보고한 선행연구[5,26,27] 및 미혼이 기혼에 비해 고위험 음주가 높게 나타난 선행연구[30]와는 상반되는 결과이다. 이러한 차이는 본 연구는 성인 남성만을 대상으로 한 반면 선행연구는 남성과 여성을 구분하지 않거나[5], 여성만을 대상[30]으로 한 연구임을 고려하면 연구대상에 의한 차이라고 할 수 있다. 남성의 결혼 상태와 고위험 음주의 관계에 대한 연구에서는 미혼 남성에 비해 기혼 남성과 별거 상태인 남성에서 고위험 음주가 높게 나타난 반면 사별과 이혼 상태인 남성에서는 고위험 음주가 낮게 나타남을 보고하였고[13], 미혼 남성과 유배우자 기혼 남성의 차이는 없는 반면 이혼 및 별거 남성에서 고위험 음주가 높게 나타남을 보고하기도 하였다[31]. 이러한 연구결과의 차이에 대해 배우자의 관심과 간섭은 음주 행위에 긍정적이거나 부정적으로 작용할 수 있기 때문이라고 하였다[13]. 불안정한 부부관계는 고위험 음주 위험을 높이는 반면 안정적인 부부관계는 음주 상황에서 보호 요인으로 작용하며[32], 기혼이나 이혼은 미혼에 비해 정신적으로 힘든 스트레스 요인이 있어서 이에 대한 해소 방법으로 음주를 택했을 가능성도 있다[23]. 따라서 추후 연구에서는 결혼 상태에 따른 고위험 음주 위험뿐 아니라 부부관계에 대한 만족도나 배우자와의 관계 등을 파악하고 이에 따른 고위험 음주 위험에 대해 파악하는 것이 필요할 것이다. 또한 배우자와 함께 거주하는 기혼 남성의 고위험 음주는 가정폭력으로 이어질 가능성이 있으며[33], 코로나19에 대한 대응책인 폐쇄와 재택 근무 등으로 인해 음주로 인한 교통사고는 감소하였으나 가정폭력이 증가한 것으로 보고되었다[3]. 따라서 배우자와 함께 거주하는 기혼 남성의 고위험 음주 실태를 파악하고 이와 관련된 문제를 관리하고 예방하기 위한 대책을 마련하는 것이 필요하다고 할 것이다.

본 연구에서 성인 음주 남성의 고위험 음주는 주말 수면시간이 7-8시간인 남성에 비해 6시간 이하인 남성에서 1.51배 많은 것으로 나타났다. 이는 주중 수면시간과 주말 수면시간의 구분없이 수면시간과 고위험 음주와의 관계를 분석한 선행연구에서 수면시간이 적은 남성에서 고위험 음주가 높게 나타난 선행연구[34]와 일치하는 결과이며, 수면시간과 고위험 음주는 관계가 없음을 보고한 선행연구[19,22]와는 차이가 있다. 음주와 수면은 상호관계가 있는 것으로 보고되고 있는데, 짧은 수면시간 등의 수면 장애는 음주에 대한 동기화를 증가시키고 고위험 음주의 가능성을 높이며[35,36], 음주량 증가는 수면장애를 초래하는 주요한 요인이다[37,38]. 따라서 본 연구 및 선행연구[34]에서 수면시간이 6시간 이하인 남성에서 고위험 음주가 높게 나타난 결과는 짧은 수면시간이 고위험 음주 위험을 높일 수 있음을 보여주는 것이라고 할 수 있다. 또한 본 연구에서는 주중 수면시간은 고위험 음주와 관계가 없었고 주말 수면시간만 고위험 음주와 관계가 있는 것으로 나타났다. 수면시간과 자살 생각에 대한 연구에서 주중 수면시간과 자살 생각은 관계가 없는 반면 주말 수면시간은 자살 생각과 관계가 있음을 보고하면서 주중에 충분한 수면을 하지 못했더라도 주말에 수면을 보완하는 경우 자살 생각에 영향을 미치지 않기 때문이라고 하였다[39]. 본 연구에서 주말 수면시간만 고위험 음주와 관련 있는 것으로 나타난 것도 같은 맥락으로 추론해 볼 수 있다. 그러나 고위험 음주 관련 선행연구들에서 수면시간과 고위험 음주의 관계를 파악한 연구는 부족한 실정이며, 실시한 연구에서도 서로 다른 결과를 보고하고 있다. 또한 코로나19 팬데믹 기간 동안 수면 양상에도 변화가 있었음을 보고하고 있으므로[10], 본 연구에서 수면시간이 짧은 남성에서 고위험 음주가 높게 나타난 결과는 수면시간이 짧기 때문일 수도 있으나 코로나19로 인해 수면시간이 감소하여 나타난 결과일 수도 있다. 따라서 수면시간과 고위험 음주의 관계에 대해서는 추후 계속적인 연구가 실시될 필요가 있으며, 주중 수면시간과 주말 수면시간으로 구분하여 비교연구를 실시하는 것이 필요하다고 할 것이다.

본 연구에서 성인 음주 남성의 고위험 음주는 스트레스 비인지 남성에 비해 스트레스를 인지하는 남성에서 1.3배 많은 것으로 나타났다. 이는 스트레스를 적게 느끼는 경우 고위험 음주 위험이 낮아짐을 보고한 선행연구[13,23]와 일치하는 결과이다. 스트레스, 슬픔, 외로움 등과 같은 부정적 감정이 있는 경우 이를 회피하거나 해소하기 위해 음주를 시작하고 지속적으로 음주를 하면서 폭음이나 과음과 같은 문제 음주를 할 위험이 높다[31]. 코로나19 팬데믹 이후 음주량이 증가했는데 이러한 음주량의 증가는 코로나19로 인한 사회적 격리, 감염에 대한 두려움, 부적절한 정보, 재정적 소실 등으로 인한 스트레스 증가와 관련 있는 것으로 보고되었으며[9,40], 전반적인 정신건강 상태가 낮은 성인에서 음주량이 증가한 것으로 보고되었다[41]. 따라서 스트레스 관리는 고위험 음주 관리 시 중요한 요인으로[30], 고위험 음주 중재 시 스트레스 정도를 파악하고 스트레스와 관련된 요인을 파악하여 스트레스를 해소하거나 감소시키기 위한 전략을 포함하는 것이 필요하다고 할 것이다.

본 연구에서 성인 음주 남성의 고위험 음주는 체질량지수가 정상인 남성에 비해 저체중인 남성에서 적은 것으로 나타났다. 이는 비만군은 정상체중군보다 고위험 음주가 1.4배 높게 나타났음을 보고한 선행연구[5] 및 체질량지수가 25 kg/m^2^ 이하인 남성에 비해 체질량지수가 25 kg/m^2^ 이상인 남성에서 고위험 음주가 높게 나타난 선행연구[19]와 같은 맥락으로 이해할 수 있다. 알코올은 고에너지 물질이며 음주는 식욕을 자극하여 음식섭취를 늘리고 지방조직 등의 기관에서 지방의 산화를 방해하여 체내 지방 축적을 증가시킨다[42]. 음주와 비만의 관계에 대한 선행연구에서도 고위험 음주가 비만 및 복부비만의 위험을 높이는 것으로 보고하였다[43], 그러나 본 연구에서와 같이 비만인 경우 고위험 음주가 높음을 보고하였고[5,19], 비만이 음주를 증가시킴을 보고하기도 하였다[44], 비만인 경우 교육, 건강, 고용 환경 등에서의 차별로 인해 자존감 저하나 우울과 같은 정서적 문제를 경험하는 경우가 많은데[45], 정서적 문제는 음주를 증가시키는 것으로 보고하고 있다[46]. 본 연구에서도 스트레스를 인지하는 남성에서 고위험 음주가 높게 나타났는데, 선행연구 및 본 연구결과를 고려하면 비만과 관련된 스트레스가 고위험 음주를 증가시켰을 것으로 추론해 볼 수 있다. 또한 비만은 폐쇄수면무호흡, 위식도역류병, 골관절염으로 인한 통증, 천식 등으로 인한 수면 장애 위험이 높은데[47], 본 연구에서 수면 부족인 남성에서 고위험 음주가 높게 나타난 결과를 고려하면 비만과 관련된 수면 문제가 고위험 음주를 증가시켰을 가능성도 있다. 따라서 남성의 고위험 음주를 관리하기 위해서는 체질량지수를 고려한 음주 관리 전략을 수립할 필요가 있으며[19], 비만 남성에서 고위험 음주가 높게 나타난 구체적인 이유를 파악하고 이에 따른 중재를 제공하는 것이 필요하다고 할 것이다. 또한 코로나19 팬데믹 이후 남성 비만이 큰 폭으로 증가하였음[11]을 고려하면, 이러한 중재는 우선적으로 제공될 필요가 있다고 할 것이다.

## 결론

본 연구는 한국 성인 음주 남성의 고위험 음주 관련 요인을 파악하기 위해 실시하였으며, 연구결과 한국 성인 음주 남성의 고위험 음주는 흡연 남성, 중졸 이하 남성, 배우자와 함께 거주하는 기혼 남성, 주말 수면시간이 6시간 이하인 남성, 스트레스를 인지하는 남성에서 높게 나타났고, 체질량지수가 저체중인 남성에서 낮게 나타났다. 따라서 성인 음주 남성의 고위험 음주를 낮추기 위해서는 이러한 고위험 음주 관련 요인을 고려한 대책을 마련하는 것이 필요하다. 특히 흡연은 고위험 음주의 주요한 요인으로 나타났으므로 음주와 흡연을 통합하여 관리하는 것이 필요하며, 배우자와 함께 거주하는 기혼 남성의 고위험 음주는 가정폭력으로 이어질 수 있으므로 이에 대한 고려가 필요하다.

본 연구는 국가단위의 조사자료인 국민건강영양조사 자료를 이용하여 성인 음주 남성의 고위험 음주 관련 요인을 파악했다는 점에서 의의가 있다. 그러나 본 연구는 코로나19 팬데믹 이후인 2020년의 자료를 이용했으므로, 본 연구결과는 코로나19 팬데믹으로 인한 일시적인 결과일 수 있다. 따라서 코로나19로 인한 일시적인 결과인지를 파악하기 위해 고위험 음주 관련 요인에 대한 반복연구를 실시하고 결과에 대한 비교분석을 계속할 것을 제언한다. 또한 남성뿐 아니라 여성의 고위험 음주율 및 고위험 음주 관련 요인을 파악하는 연구를 실시하고, 고위험 음주율 및 고위험 음주 관련 요인에서 남성과 여성의 차이를 비교하는 연구를 실시할 것을 제언한다.

## Figures and Tables

**Table 1.** High-risk Drinking according to the General Characteristics of Alcohol-drinking Men (N = 1,653)

Characteristics	Categories	High-risk drinking				Rao-Scott χ^2^ (*p*)
		Yes (n = 445)		No (n = 1,208)		
		n^†^	%^‡^ (SE)	n^†^	%^‡^ (SE)	
Age (yr)	19-29	57	15.9 (2.4)	294	84.1 (2.4)	38.45 (< .001)
	30-39	73	25.5 (2.6)	241	74.5 (2.6)	
	40-49	118	34.5 (3.0)	248	65.5 (3.0)	
	50-59	119	29.2 (2.4)	280	70.8 (2.4)	
	60-64	78	33.3 (3.6)	145	66.7 (3.6)	
Income	Low	39	23.8 (4.0)	100	76.2 (4.0)	2.02 (.650)
	Middle-low	88	27.7 (2.6)	224	72.3 (2.6)	
	Middle-high	140	28.9 (2.6)	372	71.1 (2.6)	
	High	178	26.1 (1.8)	510	73.9 (1.8)	
Education	≤ Middle school	61	41.0 (4.9)	82	59.0 (4.9)	13.99 (.006)
	High school	175	26.5 (1.9)	487	73.5 (1.9)	
	≥ University	181	24.6 (1.8)	591	75.4 (1.8)	
Marital status	Unmarried	103	18.4 (1.9)	441	81.6 (1.9)	35.15 (< .001)
	Living with spouse	299	31.5 (1.8)	705	68.5 (1.8)	
	Divorce, separation, bereavement	42	34.9 (4.9)	61	65.1 (4.9)	
Occupation	No	61	17.6 (2.4)	260	82.4 (2.4)	17.74 (< .001)
	Yes	384	29.0 (1.5)	948	71.0 (1.5)	
Household type	One-person	60	26.5 (3.2)	144	73.5 (3.2)	0.03 (.862)
	Multi-person	385	27.1 (1.4)	1064	72.9 (1.4)	
Household head	No	137	22.1 (1.8)	481	77.9 (1.8)	13.46 (.001)
	Yes	308	30.3 (1.6)	727	69.7 (1.6)	

SE = standard error. ^†^Unweighted frequency; ^‡^Weighted percentage.

**Table 2.** High-risk Drinking according to Health-related Characteristics and Health Behavior of Alcohol-drinking Men (N = 1,653)

Characteristics	Categories	High-risk drinking				Rao-Scott χ^2^ (*p*)
		Yes (n = 445)		No (n = 1,208)		
		n^†^	%^‡^ (SE)	n^†^	%^‡^ (SE)	
Body mass index	Underweight	4	7.3 (3.7)	31	92.7 (3.7)	8.73 (.036)
	Normal	99	25.5 (2.5)	281	74.5 (2.5)	
	Overweight	111	26.4 (2.3)	314	73.6 (2.3)	
	Obese	229	29.1 (1.9)	574	70.9 (1.9)	
Abdominal obesity	No	233	24.4 (1.5)	692	75.6 (1.5)	8.30 (.021)
	Yes	212	30.7 (2.2)	510	69.3 (2.2)	
Perceived health	Poor	70	29.3 (3.2)	143	70.7 (3.2)	14.65 (.001)
	Ordinary	241	29.9 (1.7)	576	70.1 (1.7)	
	Good	109	20.8 (1.9)	443	79.2 (1.9)	
Perceived stress	No	288	25.0 (1.4)	862	75.0 (1.4)	7.12 (.005)
	Yes	157	31.7 (2.2)	345	68.3 (2.2)	
Medical check-up	No	134	26.7 (2.3)	361	73.3 (2.3)	0.00 (.989)
	Yes	283	26.6 (1.6)	799	73.4 (1.6)	
Influenza	No	293	25.7 (1.6)	819	74.3 (1.6)	1.53 (.308)
vaccination	Yes	123	28.7 (2.4)	341	71.3 (2.4)	
Unmet medical	No	373	27.0 (1.4)	1035	73.0 (1.4)	0.72 (.478)
needs	Yes	19	22.7 (5.6)	59	77.3 (5.6)	
Smoking	No	217	21.3 (1.4)	806	78.7 (1.4)	44.16 (< .001)
	Yes	228	36.2 (2.2)	402	63.8 (2.2)	
Physical activities	No	230	28.4 (1.8)	592	71.6 (1.8)	2.80 (.106)
	Yes	187	24.7 (1.6)	568	75.3 (1.6)	
Walking (days/week)	< 5	255	29.4 (1.7)	643	70.6 (1.7)	8.09 (.016)
	≥ 5	162	23.0 (2.0)	517	77.0 (2.0)	
Muscle exercise (days/week)	< 2	303	28.8 (1.5)	769	71.2 (1.5)	7.76 (.010)
	≥ 2	114	22.1 (2.0)	391	77.9 (2.0)	
Sitting time (hours/day)	< 10	254	29.5 (1.9)	606	70.5 (1.9)	5.47 (.041)
	≥ 10	191	24.4 (1.7)	602	75.6 (1.7)	
Weekday sleep time (hours/day)	≤ 6	215	28.1 (1.9)	547	71.9 (1.9)	3.35 (.271)
	7~8	184	25.1 (1.7)	560	74.9 (1.7)	
	≥ 9	46	31.4 (4.2)	101	68.6 (4.2)	
Weekend sleep time (hours/day)	≤ 6	139	34.1 (3.0)	277	65.9 (3.0)	13.67 (.013)
	7~8	204	24.7 (1.7)	623	75.3 (1.7)	
	≥ 9	102	24.8 (2.5)	308	75.2 (2.5)	

SE = standard error. ^†^Unweighted frequency; ^‡^Weighted percentage.

**Table 3.** Factors associated With High-risk Drinking in Men (N = 1,653)

Factors (reference)	Categories	OR	95% CI	*p*
Age (yr, 19-29)	30-39	1.02	0.56-1.86	.959
	40-49	1.31	0.73-2.35	.361
	50-59	0.85	0.47-1.54	.595
	60-64	1.16	0.59-2.28	.663
Education (≥ University)	≤ Middle school	1.91	1.13-3.20	.016
	High school	1.18	0.88-1.58	.278
Marital status (Unmarried)	Living with spouse	1.61	1.06-2.44	.025
	Divorce, separation, bereavement	1.67	0.91-3.07	.097
Occupation (No)	Yes	1.46	0.97-2.19	.069
Household head (No)	Yes	1.13	0.84-1.52	.404
Body mass index (Normal)	Underweight	0.19	0.06-0.62	.006
	Overweight	1.01	0.69-1.46	.975
	Obese	1.16	0.71-1.90	.556
Abdominal obesity (No)	Yes	1.06	0.67-1.69	.794
Perceived health (Poor)	Good	0.87	0.58-1.26	.432
	Ordinary	1.17	0.82-1.67	.381
Perceived stress (No)	Yes	1.30	1.01-1.67	.044
Smoking (No)	Yes	2.11	1.62-2.73	<.001
Walking (≥ 5) (days/week)	< 5	1.10	0.82-1.46	.531
Muscle exercise (≥ 2) (days/week)	< 2	1.10	0.84-1.46	.488
Sitting time (≥ 10) (hours/day)	< 10	1.27	0.95-1.69	.101
Weekend sleep time (7-8) (hours/day)	≤ 6	1.51	1.08-2.12	.016
	≥ 9	1.04	0.70-1.53	.857

OR = odds ratio; CI = confidence interval.

## Data Availability

The National Health and Nutrition Examination Survey data are freely available on website (https://knhanes.kdca.go.kr/knhanes/sub03/sub03_02_05.do).
